# Prediction of survival and immunotherapy response by the combined classifier of G protein-coupled receptors and tumor microenvironment in melanoma

**DOI:** 10.1186/s40001-023-01346-6

**Published:** 2023-09-16

**Authors:** Kangjie Shen, Qiangcheng Wang, Lu Wang, Yang Yang, Min Ren, Yanlin Li, Zixu Gao, Shaoluan Zheng, Yiteng Ding, Jiani Ji, Chenlu Wei, Tianyi Zhang, Yu Zhu, Jia Feng, Feng Qin, Yanwen Yang, Chuanyuan Wei, Jianying Gu

**Affiliations:** 1 Department of Plastic Surgery, Zhongshan Hospital, Fudan University, 180 Fenglin Road, Shanghai, China; 2 Jiangsu Cancer Hospital, Jiangsu Institute of Cancer Research, The Affiliated Cancer Hospital of Nanjing Medical University, Nanjing, China; 3 Department of Plastic and Reconstructive Surgery, Zhongshan Hospital (Xiamen), Fudan University, Xiamen, China; 4 Xiamen Clinical Research Center for Cancer Therapy, Xiamen, China; 5 The First Clinical Medical College of Nanjing Medical University, Nanjing, China

**Keywords:** Melanoma, G protein-coupled receptors, Tumor microenvironment, Immunotherapy, Multi-omics, scRNA-seq, Pan-cancer

## Abstract

**Background:**

Melanoma is the deadliest form of skin tumor, and G protein-coupled receptors (GPCRs) play crucial roles in its carcinogenesis. Furthermore, the tumor microenvironment (TME) affects the overall survival (OS) and the response to immunotherapy. The combination of GPCRs and TME from a multi-omics perspective may help to predict the survival of the melanoma patients and their response to immunotherapy.

**Methods:**

Bulk-seq, single-cell RNA sequencing (scRNA-seq), gene mutations, immunotherapy responses, and clinicopathologic feature data were downloaded from public databases, and prognostic GPCRs and immune cells were screened using multiple machine learning algorithms. The expression levels of GPCRs were detected using real-time quantitative polymerase chain reaction (qPCR) in A375 and HaCaT cell lines. The GPCR–TME classifier was constructed and verified using different cohorts and multi-omics. Gene set enrichment analysis (GSEA), weighted gene co-expression network analysis (WGCNA), and tracking tumor immunophenotype (TIP) were used to identify the key biological pathways among the GPCR–TME subgroups. Then, tumor mutational burden (TMB), vital mutant genes, antigen presentation genes, and immune checkpoints were compared among the subgroups. Finally, the differences in immunotherapy response rates among the GPCR–TME subgroups were investigated.

**Results:**

A total of 12 GPCRs and five immune cell types were screened to establish the GPCR–TME classifier. No significant differences in the expression levels of the 12 GPCRs were found in the two cell lines. Patients with high GPCR score or low TME score had a poor OS; thus, the GPCR^low^/TME^high^ subgroup had the most favorable OS. The scRNA-seq result revealed that immune cells had a higher GPCR score than tumor and stromal cells. The GPCR–TME classifier acted as an independent prognostic factor for melanoma. GSEA, WGCNA, and TIP demonstrated that the GPCR^low^/TME^high^ subgroup was related to the activation and recruitment of anti-tumor immune cells and the positive regulation of the immune response. From a genomic perspective, the GPCR^low^/TME^high^ subgroup had higher TMB, and different mutant genes. Ultimately, higher expression levels of antigen presentation genes and immune checkpoints were observed in the GPCR^low^/TME^high^ subgroup, and the melanoma immunotherapy cohorts confirmed that the response rate was highest in the GPCR^low^/TME^high^ cohort.

**Conclusions:**

We have developed a GPCR–TME classifier that could predict the OS and immunotherapy response of patients with melanoma highly effectively based on multi-omics analysis.

**Supplementary Information:**

The online version contains supplementary material available at 10.1186/s40001-023-01346-6.

## Background

Melanoma is the primary cause of skin tumor-related death with increasing annual cases of morbidity and mortality [1, 2]. While patients with melanoma undergo a combination of surgery, chemotherapeutic drugs, and molecular-targeted therapeutic drugs, the effectiveness of current therapies varies among individuals [3, 4]. The emergence of immunotherapy has offered hope to patients with advanced melanoma, but its benefits are limited [5–7]. Due to the inconsistent treatment outcomes and varying response rates, considerable effort has been devoted to identifying prognostic and therapeutic biomarkers for melanoma, particularly those capable of predicting the efficacy of immunotherapy [8–10].

G protein-coupled receptors (GPCRs) are membrane protein receptors that bind chemicals in the cellular environment and activate a series of intracellular signaling pathways that ultimately lead to changes in cellular state [11–13]. GPCRs have also been implicated in carcinogenesis and metastasis [14–17] and have emerged as important targets for drug therapy due to their wide distribution in the body [18]. In addition, they play a pivotal role in shaping the tumor microenvironment (TME). Santagata et al. found that two GPCRs, CXCR4 and CXCR7, orchestrate the recruitment of immune and stromal cells [19]. In the context of melanoma, Ridky et al. demonstrated that a combination of anti-PD-1 and G-1, a G protein-coupled estrogen receptor-selective agonist, could effectively inhibit tumor growth [20]. Nevertheless, there is a noticeable absence of comprehensive and sophisticated studies delving into the role of GPCRs in melanoma. Furthermore, the precise mechanism through which GPCRs influence the TME and the response to immunotherapy in melanomas remain unknown.

The emergence of high-throughput sequencing techniques, particularly single-cell RNA sequencing (scRNA-seq), has propelled tumor research into the era of precision. The integration of scRNA-seq and bulk sequencing (bulk-seq) allows researchers to dissect the contribution of individual genes in tumorigenesis and progression, both at the tissue and single-cell levels [21–23]. In the current study, we have harnessed the power of GPCRs in conjunction with the intricate cellular landscape of the TME to construct a GPCR–TME classifier for better clinical classification and therapeutic strategies. This innovative approach enhances clinical classification and informs the development of more effective therapeutic strategies. To a significant extent, our findings address the limitations of current clinical staging methodologies and offer valuable insights for the precise management of melanoma.

## Materials and methods

### Sources of data

Gene expression and survival data of melanoma cohorts were obtained from two publicly available data sets (The Cancer Genome Atlas (TCGA)–SKCM and GSE65904). For scRNA-seq melanoma data, we utilized data from GSE189889 to visualize the GPCR score of different cell types within the TME. Pan-cancer data were downloaded from Xena [24]. Furthermore, we tested the utility of the GPCR–TME classifier as an immunotherapy predictor using data from three melanoma cohorts (GSE35640, GSE91061, and GSE145996) with available immunotherapy response data.

### Data preprocessing

For RNA-seq data, the normalization was performed using the R package ‘DESeq2’ based on the downloaded count data. For microarray data, ‘affy’ package was used for background correction and normalization. For scRNA-seq data, the ‘NormalizeData’ function of ‘Seurat’ package was used for normalization.

### Quantification of GPCRs and TME cells

The list of GPCRs was downloaded from the Molecular Signatures Database (GOMF_G_PROTEIN_COUPLED_RECEPTOR_ACTIVITY). Gene expression matrices of the melanoma cohorts were then extracted based on the 870 GPCRs. CIBERSORT enables the calculation of 22 immune cell types through a deconvolution algorithm using the bulk-seq data. Prior to applying CIBERSORT, we followed standard preprocessing steps of normalization of RNA-seq and microarray data, to ensure the comparability and reliability of our gene expression data. The enrichment scores calculated by CIBERSORT were utilized for developing the TME score [25].

### Establishment and verification of the GPCR score at bulk and single-cell level

Based on the survival data of the TCGA–SKCM cohort, we employed univariate Cox regression analysis with a bootstrap algorithm (resampling = 1000) to screen for GPCRs related to overall survival (OS). A significance threshold of *P* < 0.001 was used as the cutoff. Subsequently, we performed the least absolute shrinkage and selection operator (LASSO) regression analysis using the R package “glmnet,” to further refine the selection of prognostic GPCRs. Finally, we utilized multivariate Cox regression analysis with a bootstrap algorithm (resampling = 1000) to identify the GPCRs most correlated with OS. For comparative analysis of the included GPCRs across pan-cancers, we utilized Xiantao (www.xiantao.love/). Survival analysis of individual GPCRs in the TCGA–SKCM cohort was conducted using GEPIA [26]. To experimentally validate the expression levels of the included GPCRs, real-time quantitative polymerase chain reaction (qPCR) was employed to assess their expression in A375 and HaCaT cell lines. For result stability, we defined the bootstrap coefficient of each included GPCR as: bootstrap coefficient = $\frac{\text{coefficient}}{\text{bootstrap standard deviation}}$. The GPCR score was calculated using the formula: GPCR score = ${\sum }_{i=1}^{n}\text{bootstrap coefficient} \, \left(\text{included }{\text{GPCR}}_{i}\right) \times \text{expression level (included }{\text{GPCR}}_{i}\text{)}$. To categorize patients into low- or high-GPCR score groups, we utilized the median as the cutoff point. Differences in OS between these two GPCR score groups within the TCGA–SKCM cohort were investigated using the “survival” package. In addition, CIBERSORT was employed to analyze the differences in immune cell composition between the two groups. We extended the evaluation of the GPCR score’s prognostic impact to pan-cancer scenarios.

For the scRNA-seq data, we retained only those cells that exhibited more than 200 detected genes, less than 20% of mitochondrial genes, and fewer than 3% of red blood cell genes. Subsequently, we employed the R package “Seurat” to identify highly variable genes, perform principal component analysis, conduct graph-based clustering, and execute t-distributed stochastic neighbor embedding (t-SNE) analysis. The annotation of individual cells was based on classical marker genes. To validate the annotation of melanoma cells, we utilized the “inferCNV” package. To compute the GPCR score of each cell, we employed the “AddModuleScore” function, and the results were visualized by ‘FeaturePlot’ and ‘VlnPlot’ functions.

### Establishment of the TME score

For the TME score, we first calculated the abundance of immune cells in melanoma using CIBERSORT and obtained quantitative data from 22 immune cell types. Patients were divided into high- and low-infiltration groups based on the infiltration of each immune cell, and survival analysis was performed. Prognostic immune cells were defined as those exhibiting a different OS between the two groups. Furthermore, we utilized multivariate Cox regression analysis with a bootstrap algorithm (resampling = 1000) to calculate the bootstrap coefficient of the prognostic immune cells. The TME score was defined as: TME score = ${\sum }_{i=1}^{n}\text{bootstrap coefficient} \, \left(\text{prognostic }{\text{immune cell}}_{i}\right) \times \text{infiltration level (prognostic }{\text{immune cell}}_{i}\text{)}$. Patients were classified into low- or high-TME score groups based on the median, and a survival analysis was conducted to investigate the difference in OS between the two TME groups. Subsequently, we combined the GPCR score with the TME score to develop the GPCR–TME classifier. Melanoma patients were divided into four subgroups: GPCR^low^/TME^low^, GPCR^high^/TME^low^, GPCR^low^/TME^high^, and GPCR^high^/TME^high^ based on the median of GPCR and TME score. A survival analysis was performed to investigate the difference in OS between the four subgroups. Furthermore, we assessed the precision of the GPCR–TME classifier using the area under the curve (AUC) of 1-, 3-, and 5-year receiver operating characteristic curves (ROC) with the R packages “timeROC” and “survivalROC.”

### Robustness and independence of the GPCR–TME classifier

Survival analysis was utilized to investigate the differences in OS among the subgroups in the TCGA–SKCM cohort. In addition, Cox regression analyses were conducted to assess whether the GPCR–TME classifier could function as an independent prognostic factor for melanoma in the TCGA–SKCM cohort. These findings were further validated using the GSE65904 cohort.

### Enrichment analysis of the GPCR–TME classifier

Gene set enrichment analysis (GSEA) was conducted to elucidate potential pathways associated with the high-/low-GPCR and high-/low-TME groups. Weighted gene co-expression network analysis (WGCNA) was employed to cluster genes with similar expression profiles using an unsupervised analysis method [27, 28]. Subsequently, Metascape was utilized to explore the enrichment results of the genes within the key modules identified through WGCNA [29]. Subsequently, we employed the R package “fgsea,” to perform GSEA among the subgroups. Ultimately, we applied the tracking tumor immunophenotype (TIP) to explore the anticancer immune status of the subgroups based on the tumor immune cycle in seven stages [30].

### Decoding the GPCR–TME classifier at the genome level

Tumor mutational burden (TMB) has the potential to drive effective anti-tumor immune responses, ultimately leading to sustained clinical responses to immunotherapy [31]. We calculated the TMB of each patient in the TCGA–SKCM cohort using previously established methods [32] and compared TMB levels among the subgroups.

Genomic mutation data (mutect2) for the TCGA–SKCM was retrieved using the R package “TCGAbiolinks.” We utilized the “maftools” package to investigate and visualize the top 20 genes with the highest gene frequencies in both the GPCR^high^/TME^low^ and GPCR^low^/TME^high^ groups.

### Prediction of the immunotherapy response rate among GPCR–TME subgroups

Furthermore, we conducted a comparative analysis of the expression levels of antigen presentation genes and immune checkpoints across the subgroups. Finally, we constructed the GPCR–TME classifier for the three melanoma immunotherapy cohorts and investigated the response rate to immunotherapy in each subgroup.

### Statistical analysis

All statistical analyses were carried out using R 4.1.1, including the Student’s *t* test, Wilcoxon rank-sum test, Fisher’s’ exact test, log-rank test, and Cox regression analyses. For multiple groups comparison, the Bonferroni method was employed for multiple testing correction. The cutoff was set at *P* < 0.05 unless otherwise stated.

## Results

### Establishment of the GPCR score at the bulk-seq level

**Fig. 1:**
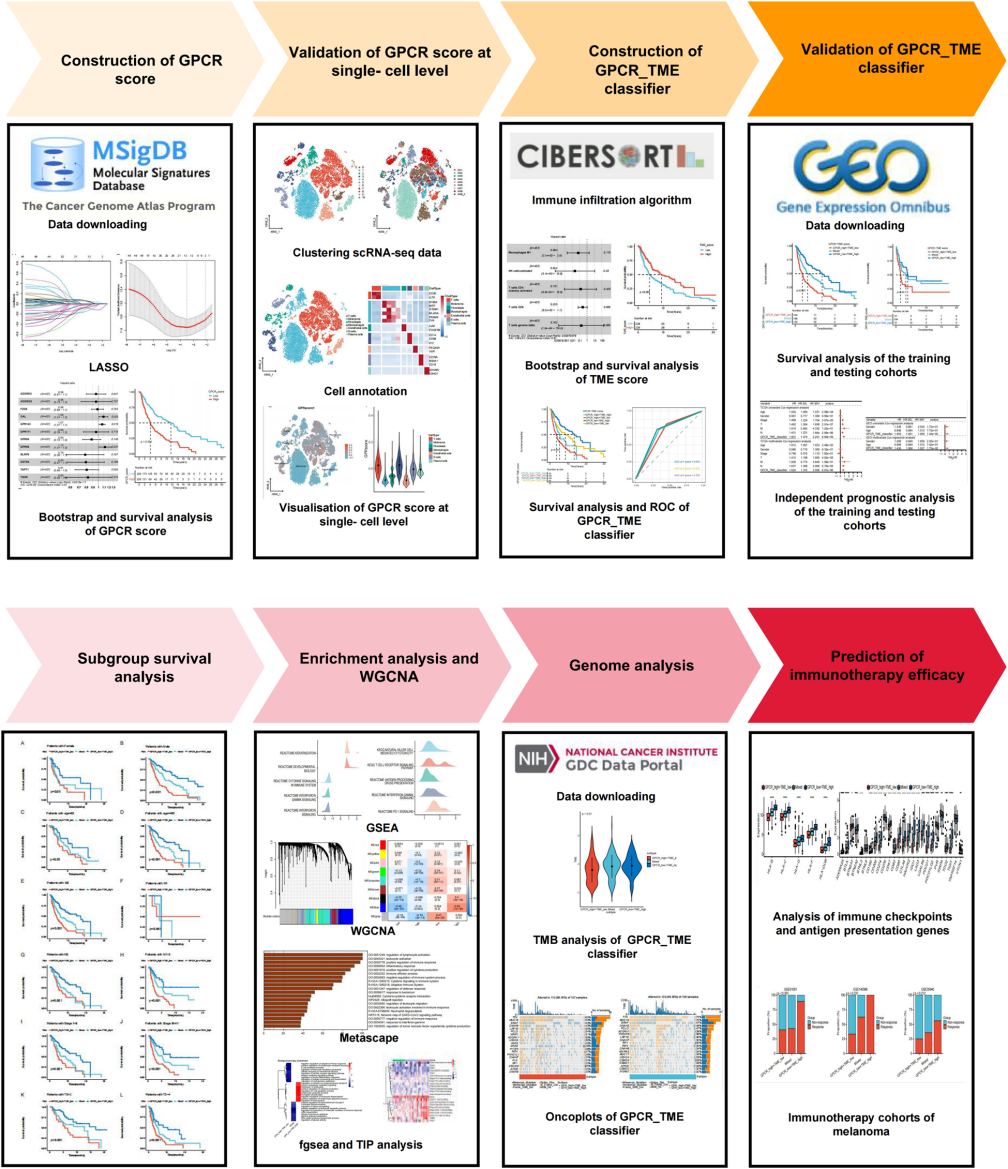
Workflow displaying the establishment and validation of GPCR–TME classifier. *GPCR* G protein-coupled receptor; *TME* Tumor microenvironment

**Fig. 2:**
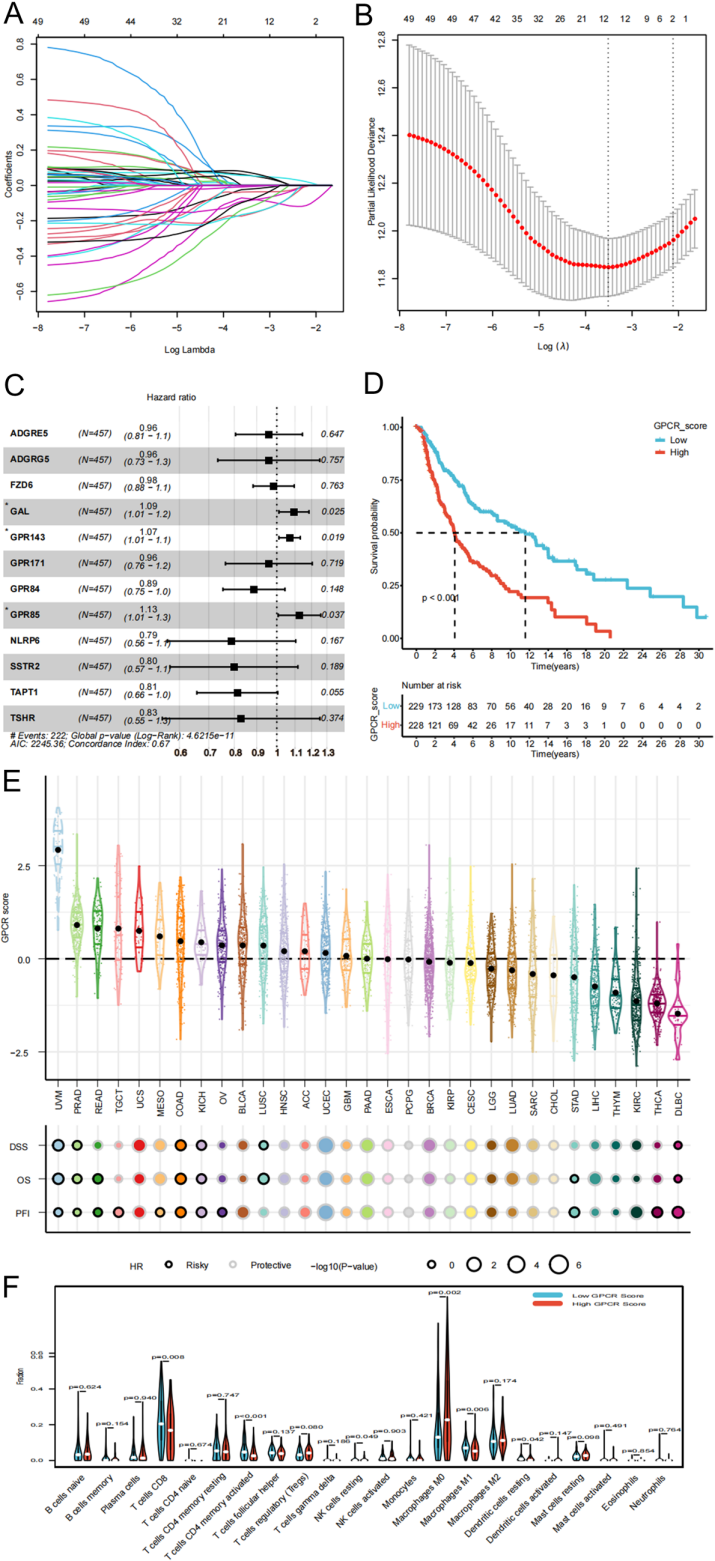
Establishment of GPCR score for melanoma and pan-cancers at bulk level. **A** Validation of prognostic GPCRs using LASSO regression analysis. **B** LASSO coefficient profile plot of prognostic GPCRs. **C** Multivariate Cox regression analysis with a bootstrap algorithm screening of 12 prognostic GPCRs. **D** Kaplan–Meier survival curves of the TCGA–SKCM cohort in patients with melanoma with high-GPCR score vs. low-GPCR score. **E** Validation of role of GPCR score in pan-cancers. **F** Decoding the different immune microenvironments of two GPCR groups using CIBERSORT. *GPCR* G protein-coupled receptor; *LASSO* least absolute shrinkage and selection operator; *TCGA* The Cancer Genome Atlas; *SKCM* Skin cutaneous melanoma

A schematic diagram is presented in Fig. 1. Additional file 8: Table S1 lists the detailed clinical information of each enrolled data set. The TCGA–SKCM cohort was utilized as the training set, and GSE65904 served as the testing set. Following univariate Cox regression analysis with the bootstrap algorithm, we identified 49 prognostic GPCRs (*P* < 0.001; Additional file 9: Table S2). Subsequently, we conducted LASSO analysis and multivariate Cox regression analyses with a bootstrap algorithm, resulting in the selection of 12 prognostic GPCRs (*P* < 0.001; Fig. 2A–C). The GPCRs’ primer sequences are provided in Additional file 10: Table S3. Owing to the ubiquity of the GPCRs, we did not find different expression levels of the 12 GPCRs in the two cell lines (Additional file 1: Figure S1). We calculated the GPCR score for the TCGA–SKCM cohort using the formula mentioned above. Patients with high-GPCR score experienced worse OS (Fig. 2D; *P* < 0.001). The GPCR score exhibited clear cancer specificity, with high score observed in uveal melanoma, prostate adenocarcinoma, and rectal adenocarcinoma, while lower score were evident in kidney renal clear cell carcinoma, thyroid carcinoma, and lymphoid neoplasm diffuse large B-cell lymphoma (Fig. 2E). Additional file 2: Figure S2 illustrates the expression levels of the 12 GPCRs across various cancer types. Additional file 3: Figure S3 shows that most of the 12 GPCRs (except GPR85 and TAPT1) were correlated with the outcomes of the melanoma. Given the potential impact of GPCRs on the TME, we employed CIBERSORT to distinguish immune infiltration levels in the TME melanoma of between the two groups (Fig. 2F; Additional file 11: Table S4). The low-GPCR score group exhibited higher levels of infiltration by CD8 T cells (*P* < 0.01), activated CD4 memory T cells (*P* < 0.001), resting natural killer (NK) cells (*P* < 0.05), M1 macrophages (*P* < 0.01), and resting dendritic cells (*P* < 0.05), while the high-GPCR score group had a higher infiltration level of M0 macrophages (*P* < 0.01).

### Verification of the GPCR scores at the single-cell level

**Fig. 3:**
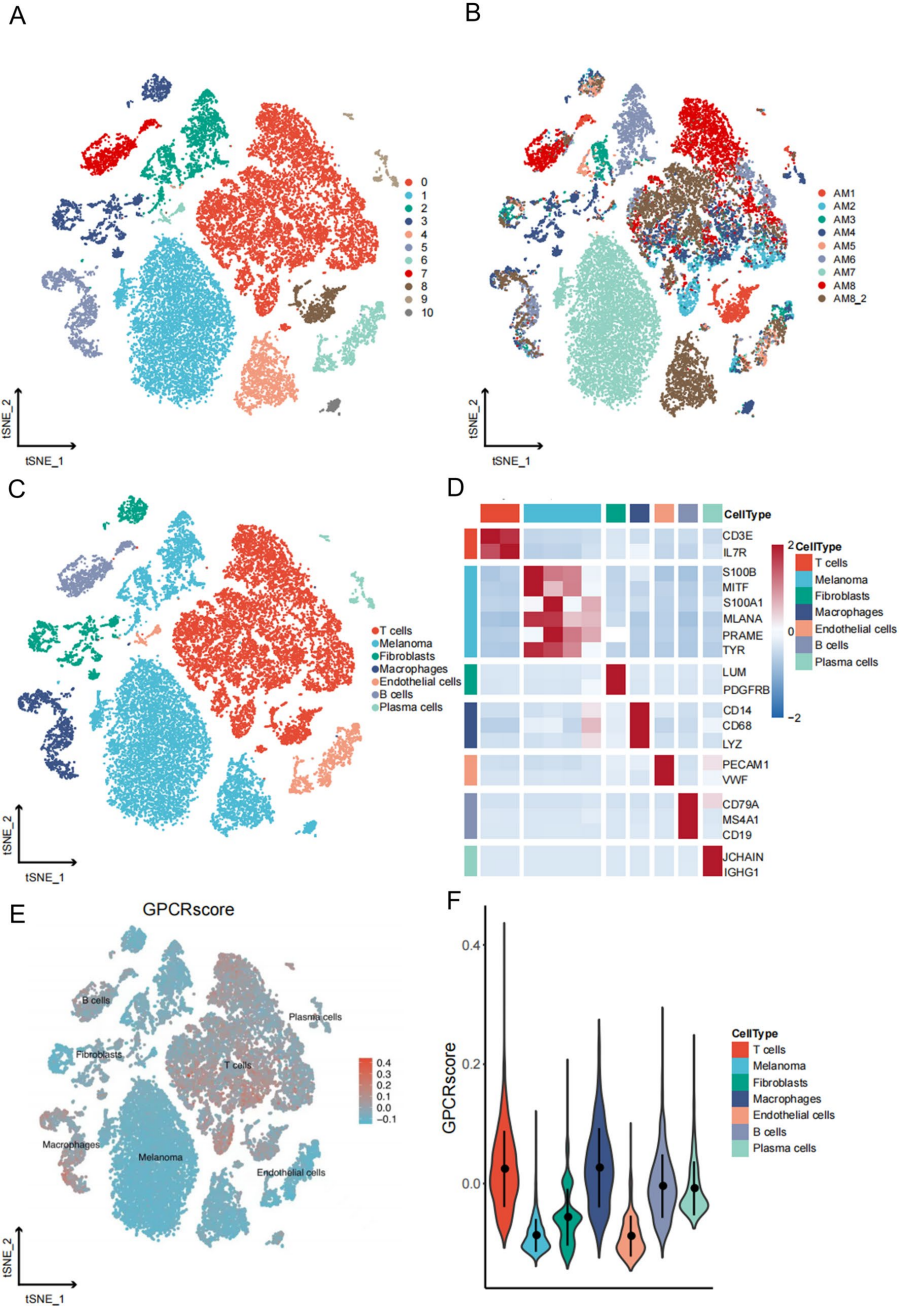
Validation of GPCR score using single-cell transcriptome analysis. **A**, **B** t-SNE plots displaying 11 clusters **A** and 9 samples **B** of melanoma. **C** t-SNE plot annotating seven cell types (T cells, melanoma, fibroblasts, macrophages, endothelial cells, B cells, and plasma cells) using classical marker genes. **D** Heatmap of marker genes used for cell type annotation. **E**, **F** Visualization of GPCR score at single-cell level using feature plot **E** and violin plot (**F**). *GPCR* G protein-coupled receptor

Furthermore, the GPCR score was validated at the single-cell transcriptomic level. Following quality control, all cells from the nine samples were grouped into 11 clusters (Fig. 3A, B). Subsequently, we identified seven cell types (T cells, melanoma, fibroblasts, macrophages, endothelial cells, B cells, and plasma cells) in the TME using established marker genes (Fig. 3C, D). The accuracy of melanoma annotation was further confirmed by the “inferCNV” method (Additional file 4: Figure S4). Feature plots were employed to visualize the expression levels of 12 GPCRs at the single-cell level (Additional file 5: Figure S5). We calculated the GPCR score of each cell by the “AddModuleScore” function, revealing that immune cells (T cells, macrophages, B cells, and plasma cells) exhibited higher GPCR score, thus confirming a relationship between immune cells and GPCRs (Fig. 3E, F).

### Establishment of the GPCR–TME classifier

**Fig. 4:**
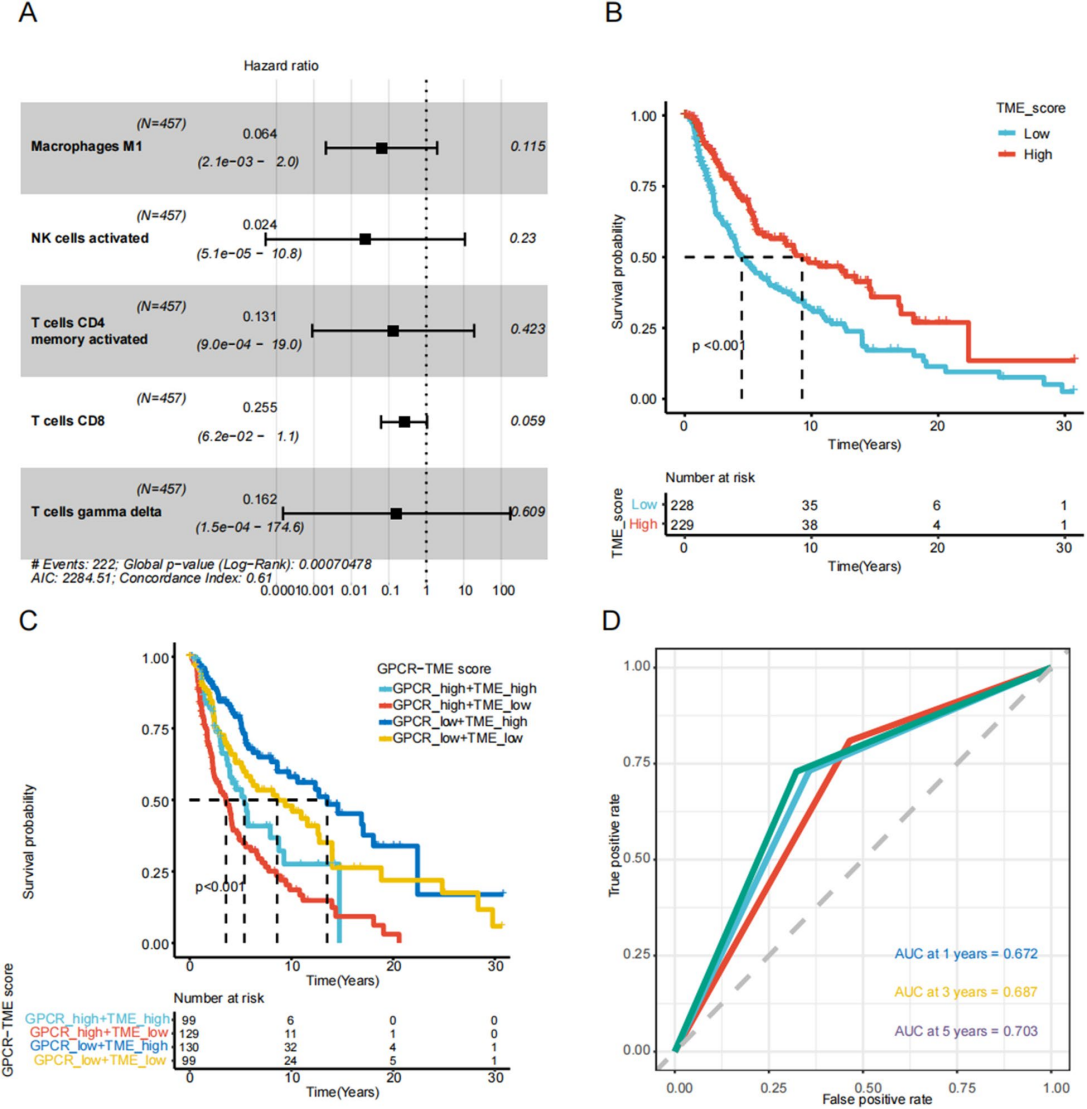
Establishment of GPCR–TME classifier. **A** Multivariate Cox regression analysis with a bootstrap algorithm screening of five types of prognostic immune cells for establishing the TME score. **B** Kaplan–Meier survival curves of TCGA–SKCM cohort in patients with melanoma high-TME vs. low-TME score. **C** Kaplan–Meier survival curves of TCGA–SKCM cohort in melanoma among the four GPCR–TME subgroups. **D** ROC curves reveal one-, three-, and five-AUC value of GPCR–TME classifier. *GPCR* G protein-coupled receptor; *TME* Tumor microenvironment; *ROC* Receiver operating characteristic curves; *AUC* Area under the curve; *TCGA* The Cancer Genome Atlas; *SKCM* Skin cutaneous melanoma

**Fig. 5:**
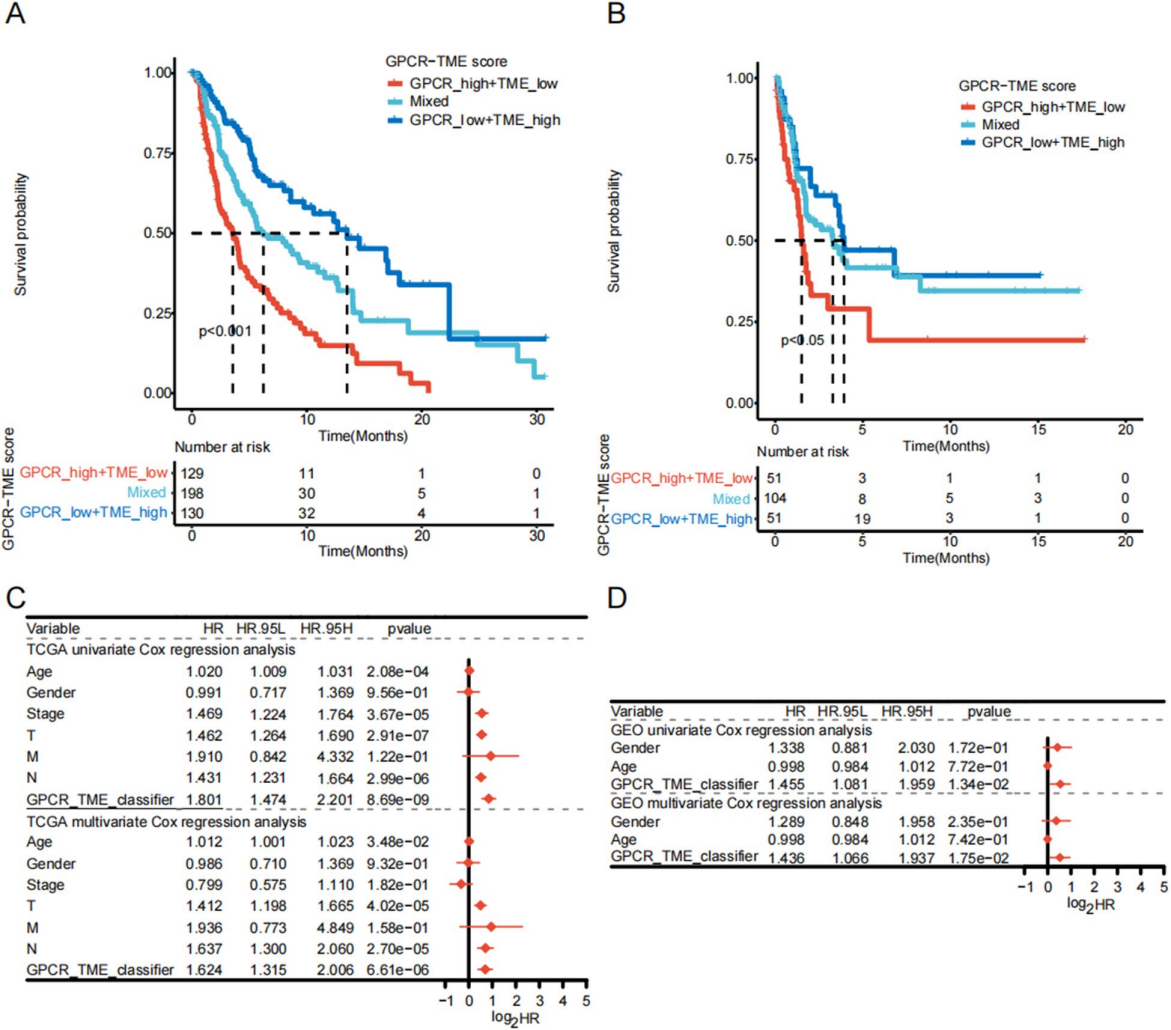
Validation of the robustness and independence of GPCR–TME classifier. **A**, **B** Kaplan–Meier survival curves of the TCGA–SKCM (**A**) and GSE65904 cohorts (**B**) in melanoma among the three GPCR–TME subgroups. **C**, **D** Independent prognostic analysis of GPCR–TME classifier in the TCGA–SKCM **C** and GSE65904 (**D**) cohorts. GPCR: G protein-coupled receptor; *TME* Tumor microenvironment; *TCGA* The Cancer Genome Atlas; *SKCM* Skin cutaneous melanoma

As the results of bulk-seq and scRNA-seq indicated potential crosstalk between the GPCRs and the immune cells, we proceeded to construct the TME score. We identified five types of immune cells associated with OS of melanoma patients (*P* < 0.05; Additional file 6: Figure S6 and Fig. 4A) and incorporated them into the construction of the TME score (Fig. 4A; Additional file 12: Table S5). Patients with higher TME scores exhibited more favorable OS (Fig. 4B; *P* < 0.001), providing a smoother transition to the subsequent analysis. We categorized patients into GPCR^low^/TME^low^, GPCR^high^/TME^low^, GPCR^low^/TME^high^, and GPCR^high^/TME^high^. Notably, patients in the GPCR^high^/TME^low^ subgroup exhibited the least favorable OS, while patients in the GPCR^low^/TME^high^ group demonstrated the most optimal OS (Fig. 4C). The GPCR–TME classifier retained its survival prediction effectiveness under different clinical characteristics (Additional file 7: Figure S7). Figure 4D demonstrates that the GPCR–TME classifier was capable of predicting 1-, 3-, and 5-year OS with a range of AUCs from 0.672 to 0.703. Considering that the OS of patients with GPCR^high^/TME^high^ and GPCR^low^/TME^low^ were less distinct, we merged GPCR^high^/TME^high^ and GPCR^low^/TME^low^ as a mixed subgroup. Survival analysis in the TCGA–SKCM and GSE65904 cohorts demonstrated that different GPCR–TME subgroups have different OS (Fig. 5A, B). Meanwhile, independent prognostic analysis showed that the GPCR–TME classifier was an independent prognostic factor for melanoma in both the TCGA–SKCM and GSE65904 cohorts (Fig. 5C, D).

### Differences in functional pathways among different GPCR–TME subgroups

**Fig. 6:**
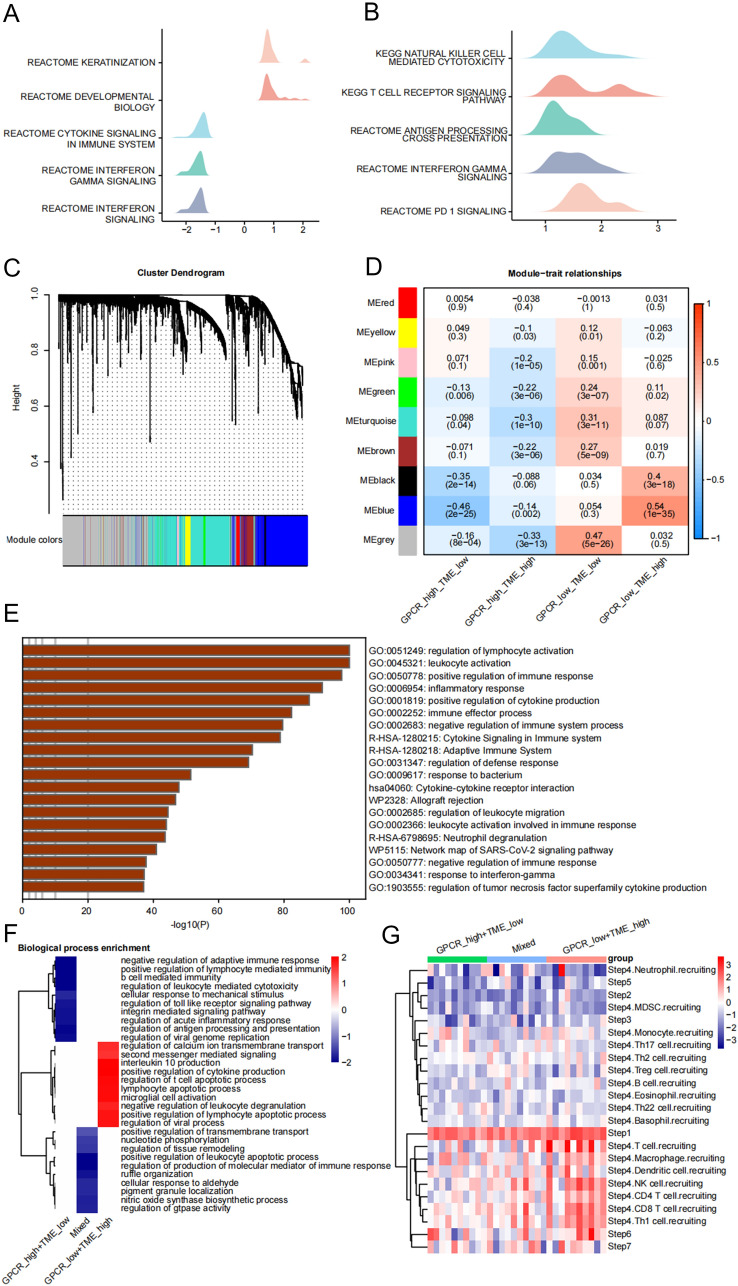
Enrichment analysis of GPCR–TME classifier. **A**, **B** GSEA of the high-/low-GPCR score groups **A** and the high-/low-TME score groups (**B**). **C** Dendrogram of clusters in which similar genes are classified into the same module. **D** Module–trait heatmap showing the blue module highly positively related to GPCR^low^/TME^high^ subgroup and negatively related to GPCR^high^/TME^low^ subgroup. **E** Enriched pathways of the blue module using Metascape. **F** fgsea showing the enriched pathways among the three GPCR–TME subgroups. **G** Analysis of anticancer immune status of the three GPCR–TME subgroups using TIP. *GSEA* Gene set enrichment analysis; GPCR: G protein-coupled receptor; TME: tumor microenvironment; *TIP* tracking tumor immunophenotype

To delve deeper into the underlying reasons for diverse prognoses among these subgroups, we conducted various enrichment analyses. The GSEA results revealed enrichment of immune system-related pathways in the low-GPCR score and high-TME score groups, while the high-GPCR score group exhibited enrichment in pathways associated with “keratinization” and “developmental biology” (Fig. 6A, B). To compare the distinct pathways among the GPCR–TME subgroups, WGCNA was performed. This analysis identified nine modules, with the blue module exhibiting a high positive correlation with the GPCR^low^/TME^high^ group and negatively related to the GPCR^high^/TME^low^; consequently, we chose it for further analysis (Fig. 6C, D). Genes in the blue module were enriched in pathways related to the positive regulation of the immune response and tumor cytotoxicity (Fig. 6E). Furthermore, fgsea revealed the enrichment of “positive regulation of cytokine production” and “interleukin 10 production” pathways within the GPCR^low^/TME^high^ subgroup (Fig. 6F). Finally, TIP analysis demonstrated that the GPCR^low^/TME^high^ subgroup was characterized by the recruitment of a majority of immune effector cells (Fig. 6G).

### Differential patterns of TMB and genomic mutation among GPCR–TME subgroups

**Fig. 7:**
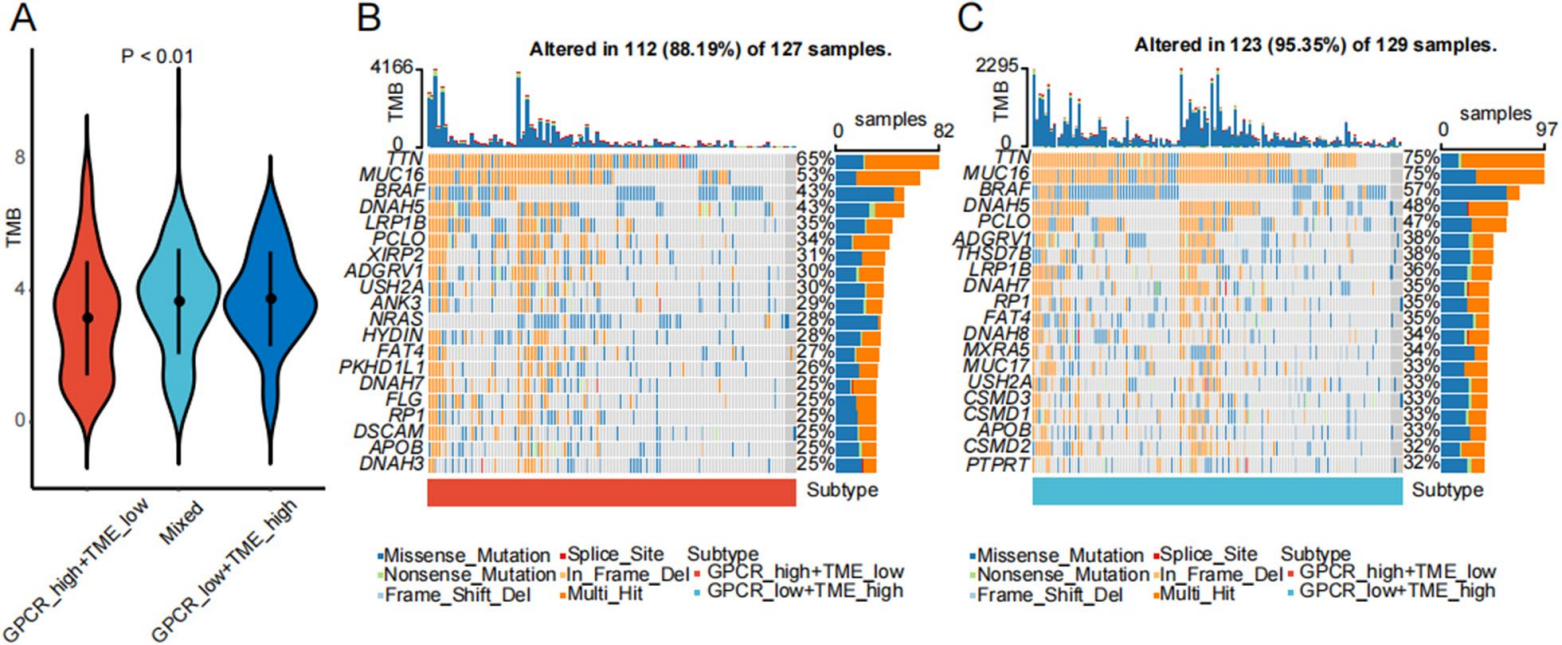
Decoding the GPCR–TME classifier at genomic level. **A** Differences of TMB in the three GPCR–TME subgroups. **B**, **C** Oncoplots showing top 20 mutant genes in the GPCR^high^/TME^low^ (**B**) and GPCR^low^/TME^high^ (**C**) subgroups in TCGA–SKCM cohort. *GPCR* G protein-coupled receptor; *TME* tumor microenvironment; *TMB* tumor mutational burden; *TCGA* the Cancer Genome Atlas; *SKCM* skin cutaneous melanoma

In comparison with the other subgroups, GPCR^low^/TME^high^ exhibited the highest TMB (Fig. 7A; *P* < 0.01). With regard to genomic mutations, we screened the top 20 variant mutations in the TCGA–SKCM cohort, and the oncoplots revealed that GPCR^low^/TME^high^ exhibited a relatively higher overall mutation rate (Fig. 7B, C). The top four mutated genes in both groups were *TTN* (65% vs. 75%), *MUC16* (53% vs. 75%), *BRAF* (43% vs. 57%), and *DNAH4* (43% vs. 48%). Regarding *TTN* and *MUC16*, multi-hit mutations were the most common mutations in both subgroups. Conversely, for *BRAF*, missense mutations prevailed in both subgroups. In the case of *DNAH4*, missense mutations were the predominant mutation in the GPCR^high^/TME^low^ subgroup, whereas multi-hit mutation were the most common mutation in GPCR^low^/TME^high^ subgroup.

### Prediction of immunotherapy response based on GPCR–TME subgroups

**Fig. 8:**
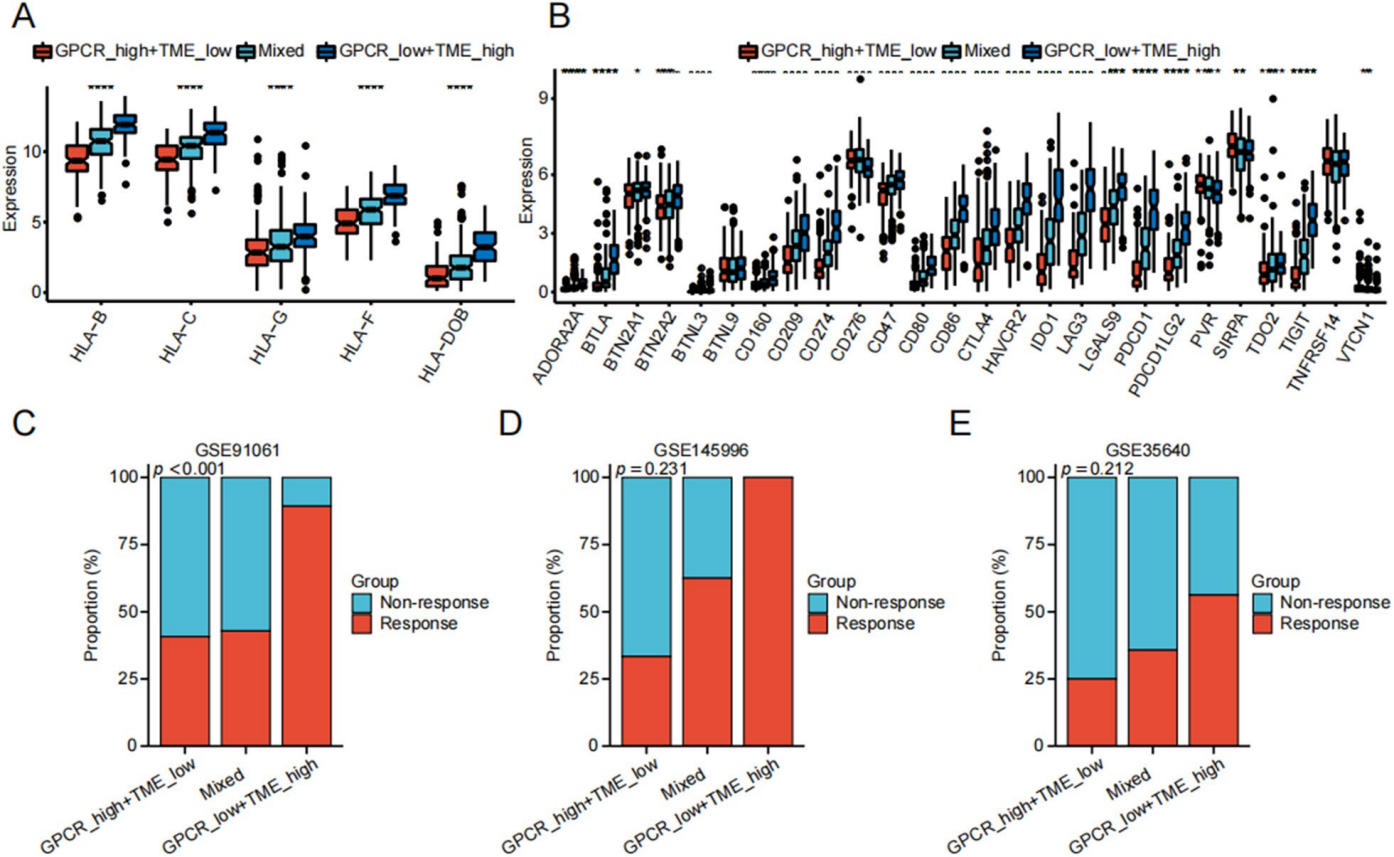
Prediction of response rate to immunotherapy using GPCR–TME classifier. **A**, **B** Expression level of antigen presentation genes (**A**) and immune checkpoints (**B**) among GPCR–TME subgroups. **C**–**E** Predicted response rate to immunotherapy in GSE91061 (**C**), GSE145996 (**D**), and GSE35640 (**E**) cohorts. *GPCR* G protein-coupled receptor; *TME* Tumor microenvironment

Given the varying immune statuses and TMB among different GPCR–TME subgroups, we hypothesized that the GPCR–TME classifier could serve as an immunotherapy predictor. The GPCR^low^/TME^high^ subgroup exhibited elevated expression levels of antigen presentation genes (Fig. 8A; all *P* < 0.001). Regarding immune checkpoints, Fig. 8B shows that expression levels of CTLA-4 (*P* < 0.001), CD274 (*P* < 0.001), PDCD1 (*P* < 0.001), TIGIT (*P* < 0.001), CD86 (*P* < 0.001), CD209 (*P* < 0.001), IDO1 (*P* < 0.001), and LAG3 (*P* < 0.001) were found to be elevated in the GPCR^low^/TME^high^ subgroup. Finally, we assessed the predictive capacity of the GPCR–TME classifier for immunotherapy response among patients treated with anti-CTLA-4, anti-PD1, or MAGE-A3 immunotherapy. The GPCR^low^/TME^high^ subgroup exhibited significantly higher immunotherapy response rate (*P* < 0.001) compared to others in the GSE91061 cohort. Though, the difference did not reach statistical significance in the GSE145996 and GSE35640 cohorts, we still observed a higher immune therapy response rate in GPCR^low^/TME^high^ group compared to others (Fig. 8D–E).

## Discussion

In recent years, the increased number of studies dedicated to GPCRs and their interaction with the TME has significantly enhanced our understanding of their critical roles in the prognosis and therapeutic approaches for cancer patients [33–36]. For instance, Zhang et al. observed a substantial down-regulation of GPRASP1 in head and neck cancers, which was notably associated with the infiltration of CD8 T cells [37]. In a separate study, Yu et al. identified GNAI2 as a risk factor for gastric cancer as it appeared to promote the accumulation of Tregs [38]. In addition, Yu et al. found that P2RY12 was downregulated in lung adenocarcinoma and exhibited a significant correlation with M2 macrophage and dendritic cell infiltration [39]. Nevertheless, these investigations primarily focused on individual GPCR for other cancer types. Studies utilizing multi-omics data, coupled with GPCRs and the TME, to predict immunotherapy response rate and OS remain relatively scarce. In our comprehensive study, we systematically integrated extensive melanoma data sets, enabling us to thoroughly explore the crosstalk between the GPCRs and the TME. The outcomes of this effort, the GPCR–TME classifier, has proven to be an exceptionally effective predictor for both the OS and immunotherapy response rate of melanoma.

Using a variety of machine learning algorithms, we identified 12 prognostic GPCRs and constructed the GPCR score. Notably, a high GPCR score signifies a poor OS for melanoma; however, it may serve as a protective factor in other cancers. This discrepancy could be attributed to the distinct roles played by these 12 GPCRs in the tumorigenesis of various cancers. Furthermore, the widespread distribution of GPCRs in vivo may account for our inability to detect differential expression levels between HaCaT and A375 cells. Considering prior research highlighting the varying impact of GPCRs on TME [40–42], we delved into the immune microenvironment of the high- and low-GPCR score groups using CIBERSORT. Our findings revealed that the low-GPCR group exhibits heightened infiltration levels of immunologic effector cells, including CD8 T cells and M1 macrophages, thus providing some insight into their improved OS.

Given that bulk-seq analysis treats all cells in the TME as homogeneous and may result in the loss of crucial information, we took a closer look at the GPCR score at the single-cell level. In the TME of melanoma, we identified seven distinct cell types, including T cells, melanoma, fibroblasts, macrophages, endothelial cells, B cells, and plasma cells. Notably, immune cells have exhibited significantly higher GPCR scores when compared to tumor and stroma cells, further substantiating the intricate relationship between GPCR and TME. Consequently, we proceeded to construct the TME score to provide a quantitative assessment of the TME in patients with melanoma. This score integrated the impact of five immune cell types that were found to be prognostically relevant in melanoma. As expected, a higher TME score demonstrated a favorable OS, and we combined the GPCR and TME scores to develop a GPCR–TME classifier. The GPCR^low^/TME^high^ subgroup had the best OS, whereas the GPCR^high^/TME^low^ subgroup had the worst OS. To further underly reasons for diverse prognoses among these subgroups, we conducted various enrichment analyses. The results shed light on the underlying mechanism of superior OS in the GPCR^low^/TME^high^ subgroup, which included the activation and recruitment of immune effector cells and the positive regulation of the immune response.

Subsequently, we sought to unravel the GPCR–TME classifier at the genomic level. Since TMB has gained widespread acceptance as a fundamental biomarker influencing responses to immunotherapy [43–45], we conducted a comparative analysis of TMB across the various GPCR–TME subgroups. Intriguingly, the GPCR^low^/TME^high^ subgroup, which had the best OS, had the highest TMB, whereas the GPCR^high^/TME^low^ subgroup displayed a significantly lower TMB. This result indicated that different GPCR–TME subgroups may have different immunotherapy response rates. In addition, we found that the mutation rate of *BRAF* was higher in the GPCR^low^/TME^high^ subgroup, implying the potential efficacy of *BRAF* inhibitors, such as vemurafenib and dabrafenib, for these patients [46, 47].

Having established that GPCRs exhibit crosstalk with the TME and that the GPCR^low^/TME^high^ subgroup exhibited higher TMB, we speculated that the GPCR–TME classifier might serve as a predictor of immunotherapy response for melanoma. Initially, we compared the expression levels of antigen presentation genes among the GPCR–TME subgroups, which all displayed upregulation in the GPCR^low^/TME^high^ subgroup. This suggests that dendritic cells may more effectively recognize tumor cells and initiate tumor eradication via CD8 T cell activation [48]. Subsequently, we investigated the expression levels of classical immune checkpoints and observed elevated levels of CTLA-4, CD274, PDCD1, TIGIT, CD86, CD209, IDO1, and LAG3 in the GPCR^low^/TME^high^ subgroup. Pul et al. found that the local delivery of anti-CTLA-4 could reduce the systemic Treg populations and activate effector T cells in melanoma [49]. CD274, also known as PD-L1, has been implicated in inducing immune evasion by tumor cells. Research by Ribas et al. demonstrated that a combination of anti-PD-L1 and dabrafenib can enhance immune infiltration and elicit a durable response in advanced melanoma [50]. Anti-PDCD1 (PD-1) therapy is a well-established immunotherapy approach for melanoma. Tjulandin et al. found that the novel PD-1 inhibitor prolgolimab could mediate significant anti-tumor effects and an endurable safety profile in advanced melanoma [51]. TIGIT is an inhibitory receptor expressed by Tregs [52]; research by Shusuke et al. suggests that the TIGIT/CD155 axis mediates resistance to ICIs in melanoma [53]. CD86 is expressed on the cell membrane of melanoma and activates the T cells, which enhances the anti-tumor effect [54]. CD209 can regulate dendritic cell trafficking and transient T-cell binding [55]; however, its role in melanoma remains unexplored. IDO1, was initially observed in plasmacytoid-shaped cells within melanoma. Kevin et al. found that IDO1 was correlated with intra-tumoral CD8 T cells and Th1-related genes, suggesting that IDO1 could act as a biomarker of immunologic tumor control [56]. LAG3 is expressed on the surface of activated CD4 and CD8 T cells [57], and Nicolas et al. found that the combination of anti-LAG-3 and anti-PD-1 enhances the cytotoxic capacity of CD8 T cells and leads to the anti-tumor effect [58]. Combining this information above, we constructed a GPCR–TME classifier for three melanoma immunotherapy cohorts. The outcomes demonstrated that the GPCR^low^/TME^high^ subgroup exhibited significantly higher immunotherapy response rate compared to others in the GSE91061 cohort. In the GSE145996 and GSE35640 cohorts, these differences did not reach statistical significance, potentially due to the bias introduced by the limited sample sizes under stringent filtering criteria. Nevertheless, it was noteworthy that we still observed a higher immune therapy response rate in GPCR^low^/TME^high^ subgroup compared to others in these two cohorts.

In a clinical application, the GPCR–TME classifier holds the potential to enhance the refinement of molecular subtyping and treatment strategies for melanoma. Specifically, following the surgical removal of a patient’s melanoma specimen, bulk-seq can be employed. Based on the gene expression data, calculations can be made to determine both the GPCR score and TME score. These scores aid in categorizing the patient into a specific GPCR–TME subgroup, enabling the prediction of the patient’s OS and their potential response rate to immunotherapy.

We acknowledge several limitations in our study. First, due to constraints related to tumor specimens, our research primarily relies on bioinformatics. We anticipate that future experiments, such as flow cytometry and immunohistochemistry, will help validate our findings. Second, to enhance the robustness of our conclusions, we recommend utilizing an internal cohort that includes gene expression data, survival data, and immunotherapy response data to further assess the performance of the GPCR–TME classifier.

## Conclusions

In summary, the multi-omics validation supports the GPCR–TME classifier as a highly effective predictor for melanoma OS and immunotherapy response rate.

### Supplementary Information

**Additional file 1: Figure S1.** Expression levels of 12 GPCRs in A375 and HaCaT cell lines. GPCR: G protein-coupled receptor.

**Additional file 2: Figure S2**. Expression levels of 12 GPCRs in pan-cancers. **A** ADGRE5. **B** ADGRG5. **C** FZD6. **D** GAL. **E** GPCR84. **F** GPCR85. **G** GPCR143. **H** GPCR171. **I** NLRP6. **J** SSTR2. **K** TAPT1. **L** TSHR. GPCR: G protein-coupled receptor.

**Additional file 3: Figure S3**. Survival analysis of single GPCR in the TCGA–SKCM cohort. **A** ADGRE5; **B** ADGRG5; **C** FZD6; **D** GAL; **E** GPR84; **F** GPR85; **G** GPR143; **H** GPR171; **I** NLRP6; **J** SSTR2; **K** TAPT1; **L** TSHR. GPCR: G protein-coupled receptor; TME: tumor microenvironment; TCGA: The Cancer Genome Atlas; SKCM: Skin cutaneous melanoma.

**Additional file 4: Figure S4**. Inferred large-scale copy number variations to identify malignant cells.

**Additional file 5: Figure S5**. Feature plots showing the expression levels of 12 GPCRs at single-cell level.

**Additional file 6: Figure S6**. Survival analysis of the prognostic immune cell types. **A** M1 macrophages. **B** Activated NK cells. **C** Activated CD4 memory T cells. **D** CD8 T cells. **E** gamma delta T cells.

**Additional file 7: Figure S7**. Subgroup survival analysis. **A** Female. **B** Male. **C** Age > 65. **D** Age ≤ 65. **E** M0. **F** M1. **G** N0. **H** N1–3. **I** Stage I−II. **J** Stages III−IV. **K** T0–2. **L** T3−4.

**Additional file 8: Table S1.** Clinical information of the included melanoma cohorts.

**Additional file 9: Table S2.** Forty-nine prognostic GPCRs identified by the univariate Cox regression analysis with bootstrap algorithm.

**Additional file 10: Table S3.** GPCRs' primer sequences.

**Additional file 11: Table S4.** Result of CIBERSORT of TCGA–SKCM cohort.

**Additional file 12: Table S5.** Five types of immune cell related to the prognosis of melanoma.

## Data Availability

The data sets generated and/or analysed during the current study are available in the TCGA and GEO databases.

## Abbreviations

AUC: Area under the curve
Bulk-seq: Bulk sequencing
GPCRs: G protein-coupled receptors
GSEA: Gene set enrichment analysis
LASSO: Least absolute shrinkage and selection operator
OS: Overall survival
qPCR: Quantitative polymerase chain reaction
ROC: Receiver operating characteristic curves
scRNA-seq: Single-cell RNA sequencing
TIP: Tracking Tumor Immunophenotype
TMB: Tumor mutational burden
TME: Tumor microenvironment
t-SNE: T-distributed stochastic neighbor embedding
WGCNA: Weighted gene co-expression network analysis

## References

[1] Siegel RL, Miller KD, Fuchs HE, Jemal A (2022). Cancer statistics, 2022. CA Cancer J Clin.

[2] Sung H, Ferlay J, Siegel RL, Laversanne M, Soerjomataram I, Jemal A (2021). Global cancer statistics 2020: GLOBOCAN estimates of incidence and mortality worldwide for 36 cancers in 185 countries. CA Cancer J Clin.

[3] Atkins MB, Lee SJ, Chmielowski B, Tarhini AA, Cohen GI, Truong T-G, et al. Combination dabrafenib and trametinib versus combination nivolumab and ipilimumab for patients with advanced -mutant melanoma: The DREAMseq Trial - ECOG-ACRIN EA6134. J Clin Oncol. 2022; 101200JCO2201763. 10.1200/JCO.22.01763 PMC9839305 36166727

[4] Zhou L, Yang Y, Si L, Chi Z, Sheng X, Lian B (2022). Phase II study of apatinib combined with temozolomide in patients with advanced melanoma after failure of immunotherapy. Melanoma Res.

[5] Vavolizza RD, Petroni GR, Mauldin IS, Chianese-Bullock KA, Olson WC, Smith KT (2022). Phase I/II clinical trial of a helper peptide vaccine plus PD-1 blockade in PD-1 antibody-naïve and PD-1 antibody-experienced patients with melanoma (MEL64). J Immunother Cancer.

[6] Larkin J, Weber J, Del Vecchio M, Gogas H, Arance AM, Dalle S (2022). Adjuvant nivolumab versus ipilimumab (CheckMate 238 trial): Reassessment of 4-year efficacy outcomes in patients with stage III melanoma per AJCC-8 staging criteria. Eur J Cancer.

[7] Reijers ILM, Menzies AM, van Akkooi ACJ, Versluis JM, van den Heuvel NMJ, Saw RPM (2022). Personalized response-directed surgery and adjuvant therapy after neoadjuvant ipilimumab and nivolumab in high-risk stage III melanoma: the PRADO trial. Nat Med.

[8] Shen K, Wang H, Xue S, Wang L, Ren M, Gao Z (2022). Genome-wide screening and immune landscape suggest a potential-m6A-related lncRNA risk signature for predicting prognosis of melanoma. Ann Transl Med.

[9] Gao Z, Wang L, Song Z, Ren M, Yang Y, Li J (2022). Intratumoral CD73: an immune checkpoint shaping an inhibitory tumor microenvironment and implicating poor prognosis in Chinese melanoma cohorts. Front Immunol.

[10] Song B, Chi H, Peng G, Song Y, Cui Z, Zhu Y (2022). Characterization of coagulation-related gene signature to predict prognosis and tumor immune microenvironment in skin cutaneous melanoma. Front Oncol.

[11] Audet M, Bouvier M (2012). Restructuring G-protein- coupled receptor activation. Cell.

[12] Venkatakrishnan AJ, Deupi X, Lebon G, Tate CG, Schertler GF, Babu MM (2013). Molecular signatures of G-protein-coupled receptors. Nature.

[13] Rosenbaum DM, Rasmussen SGF, Kobilka BK (2009). The structure and function of G-protein-coupled receptors. Nature.

[14] Mo Z, Liu M, Yang F, Luo H, Li Z, Tu G (2013). GPR30 as an initiator of tamoxifen resistance in hormone-dependent breast cancer. Breast Cancer Res.

[15] Chimento A, De Luca A, Nocito MC, Avena P, La Padula D, Zavaglia L (2020). Role of GPER-mediated signaling in testicular functions and tumorigenesis. Cells.

[16] Natale CA, Li J, Pitarresi JR, Norgard RJ, Dentchev T, Capell BC (2020). Pharmacologic activation of the G protein-coupled estrogen receptor inhibits pancreatic ductal adenocarcinoma. Cell Mol Gastroenterol Hepatol.

[17] Chan Y-T, Lai ACY, Lin R-J, Wang Y-H, Wang Y-T, Chang W-W (2020). GPER-induced signaling is essential for the survival of breast cancer stem cells. Int J Cancer.

[18] Hauser AS, Attwood MM, Rask-Andersen M, Schiöth HB, Gloriam DE (2017). Trends in GPCR drug discovery: new agents, targets and indications. Nat Rev Drug Discov.

[19] Santagata S, Ieranò C, Trotta AM, Capiluongo A, Auletta F, Guardascione G (2021). CXCR4 and CXCR7 signaling pathways: a focus on the cross-talk between cancer cells and tumor microenvironment. Front Oncol.

[20] Natale CA, Li J, Zhang J, Dahal A, Dentchev T, Stanger BZ (2018). Activation of G protein-coupled estrogen receptor signaling inhibits melanoma and improves response to immune checkpoint blockade. Elife.

[21] Song G, Shi Y, Meng L, Ma J, Huang S, Zhang J (2022). Single-cell transcriptomic analysis suggests two molecularly subtypes of intrahepatic cholangiocarcinoma. Nat Commun.

[22] Wu Y, Yang S, Ma J, Chen Z, Song G, Rao D (2022). Spatiotemporal immune landscape of colorectal cancer liver metastasis at single-cell level. Cancer Discov.

[23] Liu Y, Zhang Q, Xing B, Luo N, Gao R, Yu K (2022). Immune phenotypic linkage between colorectal cancer and liver metastasis. Cancer Cell.

[24] Goldman MJ, Craft B, Hastie M, Repečka K, McDade F, Kamath A (2020). Visualizing and interpreting cancer genomics data via the Xena platform. Nat Biotechnol.

[25] Newman AM, Liu CL, Green MR, Gentles AJ, Feng W, Xu Y (2015). Robust enumeration of cell subsets from tissue expression profiles. Nat Methods.

[26] Tang Z, Li C, Kang B, Gao G, Li C, Zhang Z (2017). GEPIA: a web server for cancer and normal gene expression profiling and interactive analyses. Nucleic Acids Res.

[27] Costanzo M, VanderSluis B, Koch EN, Baryshnikova A, Pons C, Tan G (2016). A global genetic interaction network maps a wiring diagram of cellular function. Science.

[28] Giulietti M, Occhipinti G, Principato G, Piva F (2016). Weighted gene co-expression network analysis reveals key genes involved in pancreatic ductal adenocarcinoma development. Cell Oncol.

[29] Zhou Y, Zhou B, Pache L, Chang M, Khodabakhshi AH, Tanaseichuk O (2019). Metascape provides a biologist-oriented resource for the analysis of systems-level datasets. Nat Commun.

[30] Xu L, Deng C, Pang B, Zhang X, Liu W, Liao G (2018). TIP: a web server for resolving tumor immunophenotype profiling. Cancer Res.

[31] Anagnostou V, Bardelli A, Chan TA, Turajlic S (2022). The status of tumor mutational burden and immunotherapy. Nat Cancer.

[32] Meléndez B, Van Campenhout C, Rorive S, Remmelink M, Salmon I, D'Haene N (2018). Methods of measurement for tumor mutational burden in tumor tissue. Transl Lung Cancer Res.

[33] Xiong Y, Ke R, Zhang Q, Lan W, Yuan W, Chan KNI (2022). Small activating RNA modulation of the G protein-coupled receptor for cancer treatment. Adv Sci.

[34] Lyu S, Zhang X, Tu Z, Zhou H, Ke X, Qu Y (2022). GPR108 is required for gambogic acid inhibiting NF-κB signaling in cancer. Pharmacol Res.

[35] Li T, Qiao T (2022). Unraveling tumor microenvironment of small-cell lung cancer: Implications for immunotherapy. Semin Cancer Biol.

[36] Sherman MH, Beatty GL (2022). Tumor microenvironment in pancreatic cancer pathogenesis and therapeutic resistance. Annu Rev Pathol.

[37] Zhang T, Liu G, Zhang J, Chen S, Deng Z, Xie M (2022). GPRASP1 is a candidate anti-oncogene and correlates with immune microenvironment and immunotherapeutic efficiency in head and neck cancer. J Oral Pathol Med.

[38] Yu H, Liu S, Wu Z, Gao F (2022). GNAI2 is a risk factor for gastric cancer: study of tumor microenvironment (TME) and establishment of immune risk score (IRS). Oxid Med Cell Longev.

[39] Yu L, Cao S, Li J, Han B, Zhong H, Zhong R (2021). Prognostic value and immune infiltration of a novel stromal/immune score-related P2RY12 in lung adenocarcinoma microenvironment. Int Immunopharmacol.

[40] Gentile S, Eskandari N, Rieger MA, Cuevas BD (2021). MEKK1 regulates chemokine expression in mammary fibroblasts: implications for the breast tumor microenvironment. Front Oncol.

[41] Dasgupta S, Ghosh T, Dhar J, Bhuniya A, Nandi P, Das A (2021). RGS5-TGFβ-Smad2/3 axis switches pro- to anti-apoptotic signaling in tumor-residing pericytes, assisting tumor growth. Cell Death Differ.

[42] Wiley SZ, Sriram K, Salmerón C, Insel PA (2019). GPR68: an emerging drug target in cancer. Int J Mol Sci.

[43] Sholl LM, Hirsch FR, Hwang D, Botling J, Lopez-Rios F, Bubendorf L (2020). The promises and challenges of tumor mutation burden as an immunotherapy biomarker: a perspective from the international association for the study of lung cancer pathology committee. J Thorac Oncol.

[44] Chan TA, Yarchoan M, Jaffee E, Swanton C, Quezada SA, Stenzinger A (2019). Development of tumor mutation burden as an immunotherapy biomarker: utility for the oncology clinic. Ann Oncol.

[45] Cao J, Yang X, Chen S, Wang J, Fan X, Fu S (2022). The predictive efficacy of tumor mutation burden in immunotherapy across multiple cancer types: a meta-analysis and bioinformatics analysis. Transl Oncol.

[46] Dummer R, Queirolo P, Abajo Guijarro AM, Hu Y, Wang D, de Azevedo SJ (2022). Atezolizumab, vemurafenib, and cobimetinib in patients with melanoma with CNS metastases (TRICOTEL): a multicentre, open-label, single-arm, phase 2 study. Lancet Oncol.

[47] Dummer R, Long GV, Robert C, Tawbi HA, Flaherty KT, Ascierto PA (2022). Randomized phase III trial evaluating spartalizumab plus dabrafenib and trametinib for V600-mutant unresectable or metastatic melanoma. J Clin Oncol.

[48] Jhunjhunwala S, Hammer C, Delamarre L (2021). Antigen presentation in cancer: insights into tumour immunogenicity and immune evasion. Nat Rev Cancer.

[49] van Pul KM, Notohardjo JCL, Fransen MF, Koster BD, Stam AGM, Chondronasiou D (2022). Local delivery of low-dose anti-CTLA-4 to the melanoma lymphatic basin leads to systemic T reduction and effector T cell activation. Sci Immunol.

[50] Ribas A, Algazi A, Ascierto PA, Butler MO, Chandra S, Gordon M (2020). PD-L1 blockade in combination with inhibition of MAPK oncogenic signaling in patients with advanced melanoma. Nat Commun.

[51] Tjulandin S, Demidov L, Moiseyenko V, Protsenko S, Semiglazova T, Odintsova S (2021). Novel PD-1 inhibitor prolgolimab: expanding non-resectable/metastatic melanoma therapy choice. Eur J Cancer.

[52] Chauvin J-M, Pagliano O, Fourcade J, Sun Z, Wang H, Sander C (2015). TIGIT and PD-1 impair tumor antigen-specific CD8^+^ T cells in melanoma patients. J Clin Invest.

[53] Kawashima S, Inozume T, Kawazu M, Ueno T, Nagasaki J, Tanji E (2021). TIGIT/CD155 axis mediates resistance to immunotherapy in patients with melanoma with the inflamed tumor microenvironment. J Immunother Cancer.

[54] Yuan S-M, Li H, Yang M, Zha H, Sun H, Li X-R (2015). High intensity focused ultrasound enhances anti-tumor immunity by inhibiting the negative regulatory effect of miR-134 on CD86 in a murine melanoma model. Oncotarget.

[55] Geijtenbeek TBH, Engering A, Van Kooyk Y (2002). DC-SIGN, a C-type lectin on dendritic cells that unveils many aspects of dendritic cell biology. J Leukoc Biol.

[56] Lynch KT, Gradecki SE, Kwak M, Meneveau MO, Wages NA, Gru AA (2021). IDO1 expression in melanoma metastases is low and associated with improved overall survival. Am J Surg Pathol.

[57] Kisielow M, Kisielow J, Capoferri-Sollami G, Karjalainen K (2005). Expression of lymphocyte activation gene 3 (LAG-3) on B cells is induced by T cells. Eur J Immunol.

[58] Gestermann N, Saugy D, Martignier C, Tillé L, Fuertes Marraco SA, Zettl M (2020). LAG-3 and PD-1+LAG-3 inhibition promote anti-tumor immune responses in human autologous melanoma/T cell co-cultures. Oncoimmunology.

