# Supplementary figures and images for: Prediction of survival and immunotherapy response by the combined classifier of G protein-coupled receptors and tumor microenvironment in melanoma
Source: Eur J Med Res. 2023 Sep 16;28:352. doi: 10.1186/s40001-023-01346-6 (PMC10504724; doi:10.1186/s40001-023-01346-6)

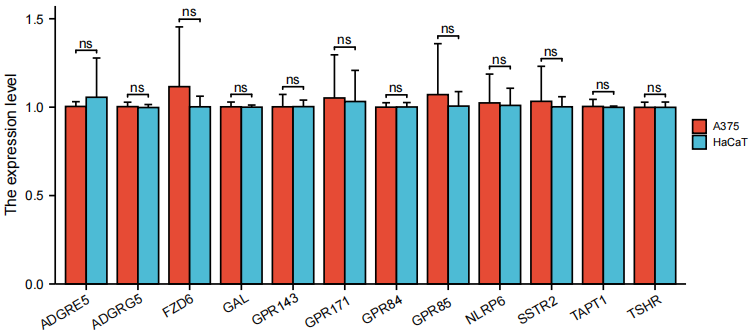

Supplement: Supplementary file 1 — Additional file 1: Figure S1. Expression levels of 12 GPCRs in A375 and HaCaT cell lines. GPCR: G protein-coupled receptor. [file 40001_2023_1346_MOESM1_ESM.tif]

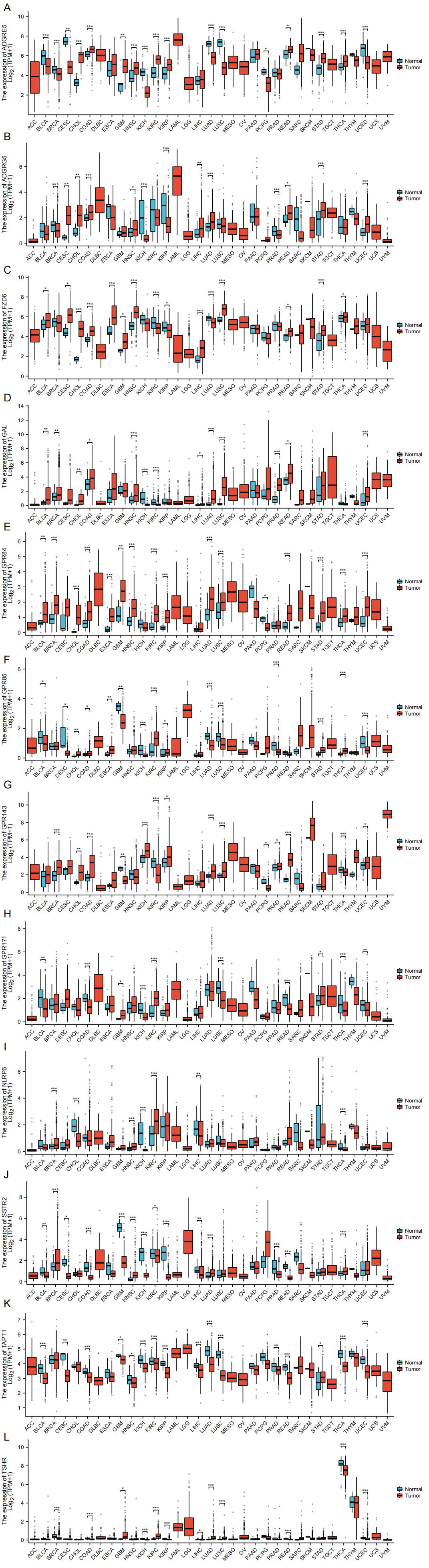

Supplement: Supplementary file 2 — Additional file 2: Figure S2. Expression levels of 12 GPCRs in pan-cancers. A ADGRE5. B ADGRG5. C FZD6. D GAL. E GPCR84. F GPCR85. G GPCR143. H GPCR171. I NLRP6. J SSTR2. K TAPT1. L TSHR. GPCR: G protein-coupled receptor. [file 40001_2023_1346_MOESM2_ESM.tif]

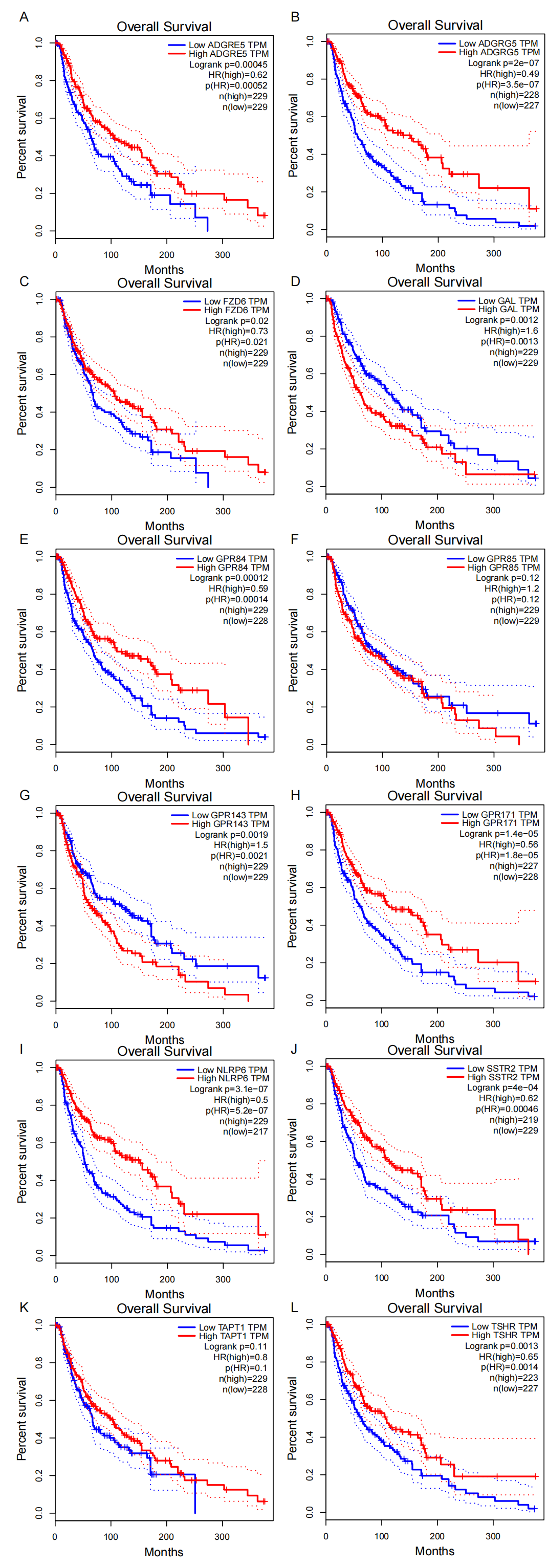

Supplement: Supplementary file 3 — Additional file 3: Figure S3. Survival analysis of single GPCR in the TCGA–SKCM cohort. A ADGRE5; B ADGRG5; C FZD6; D GAL; E GPR84; F GPR85; G GPR143; H GPR171; I NLRP6; J SSTR2; K TAPT1; L TSHR. GPCR: G protein-coupled receptor; TME: tumor microenvironment; TCGA: The Cancer Genome Atlas; SKCM: Skin cutaneous melanoma. [file 40001_2023_1346_MOESM3_ESM.tif]

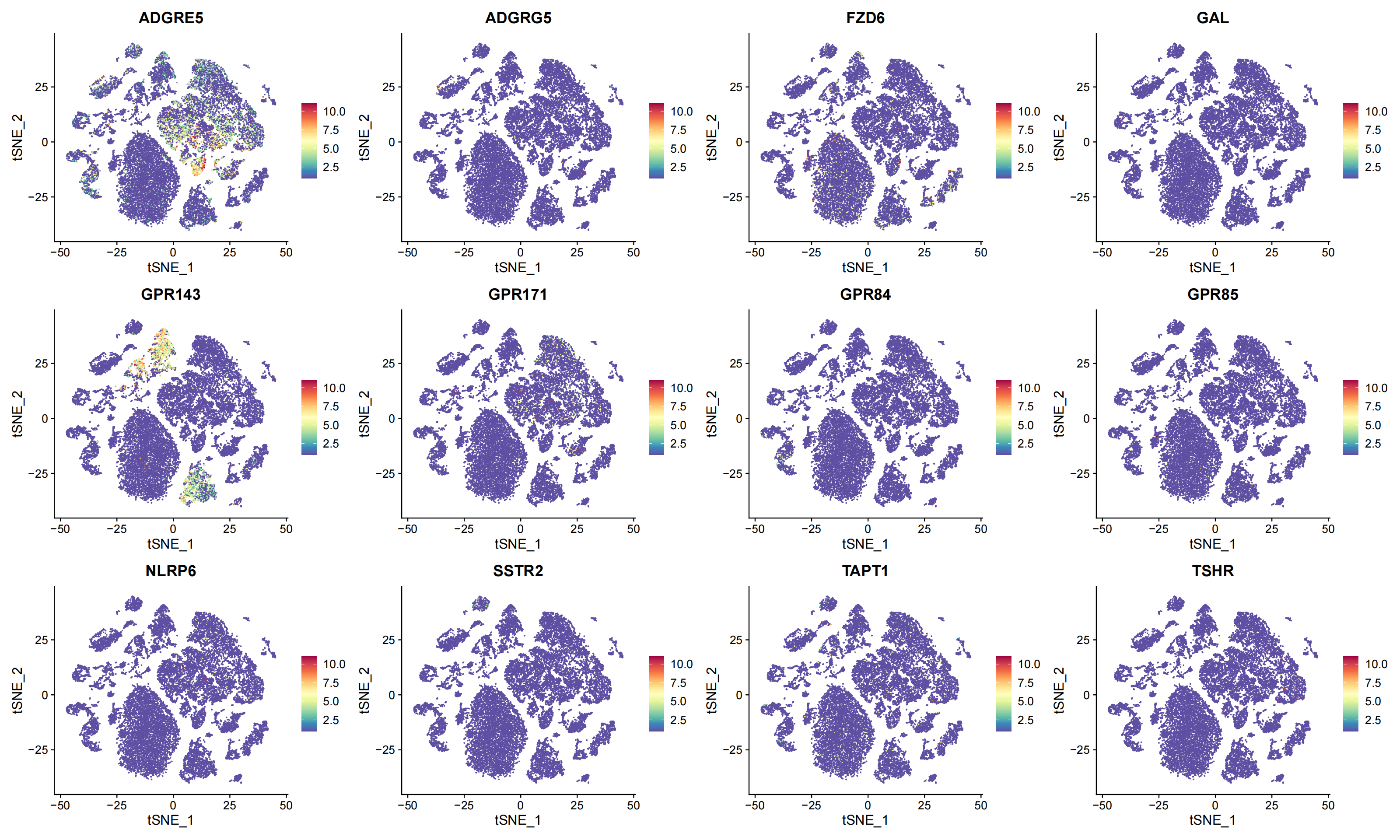

Supplement: Supplementary file 5 — Additional file 5: Figure S5. Feature plots showing the expression levels of 12 GPCRs at single-cell level. [file 40001_2023_1346_MOESM5_ESM.tif]

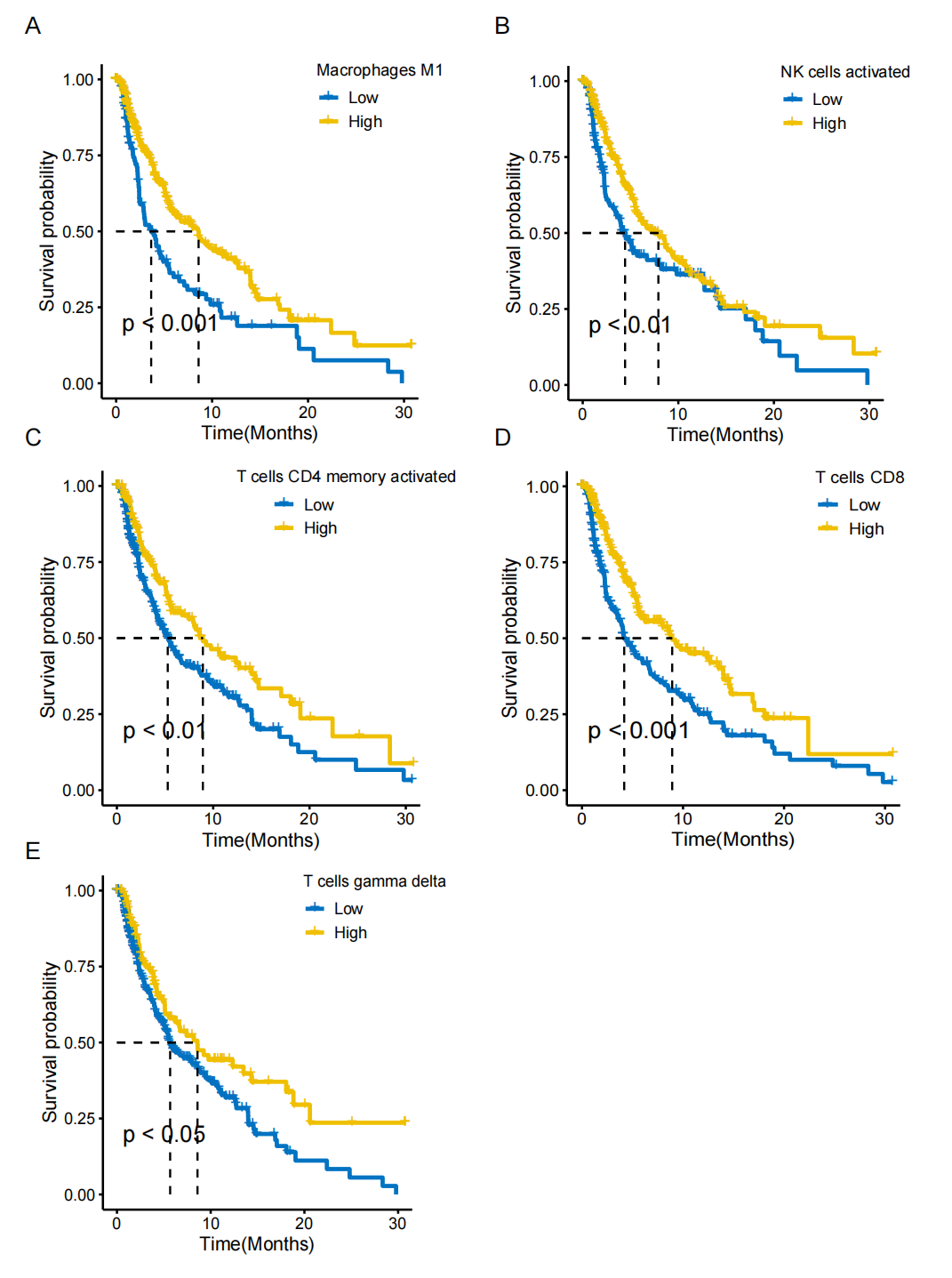

Supplement: Supplementary file 6 — Additional file 6: Figure S6. Survival analysis of the prognostic immune cell types. A M1 macrophages. B Activated NK cells. C Activated CD4 memory T cells. D CD8 T cells. E gamma delta T cells. [file 40001_2023_1346_MOESM6_ESM.tif]

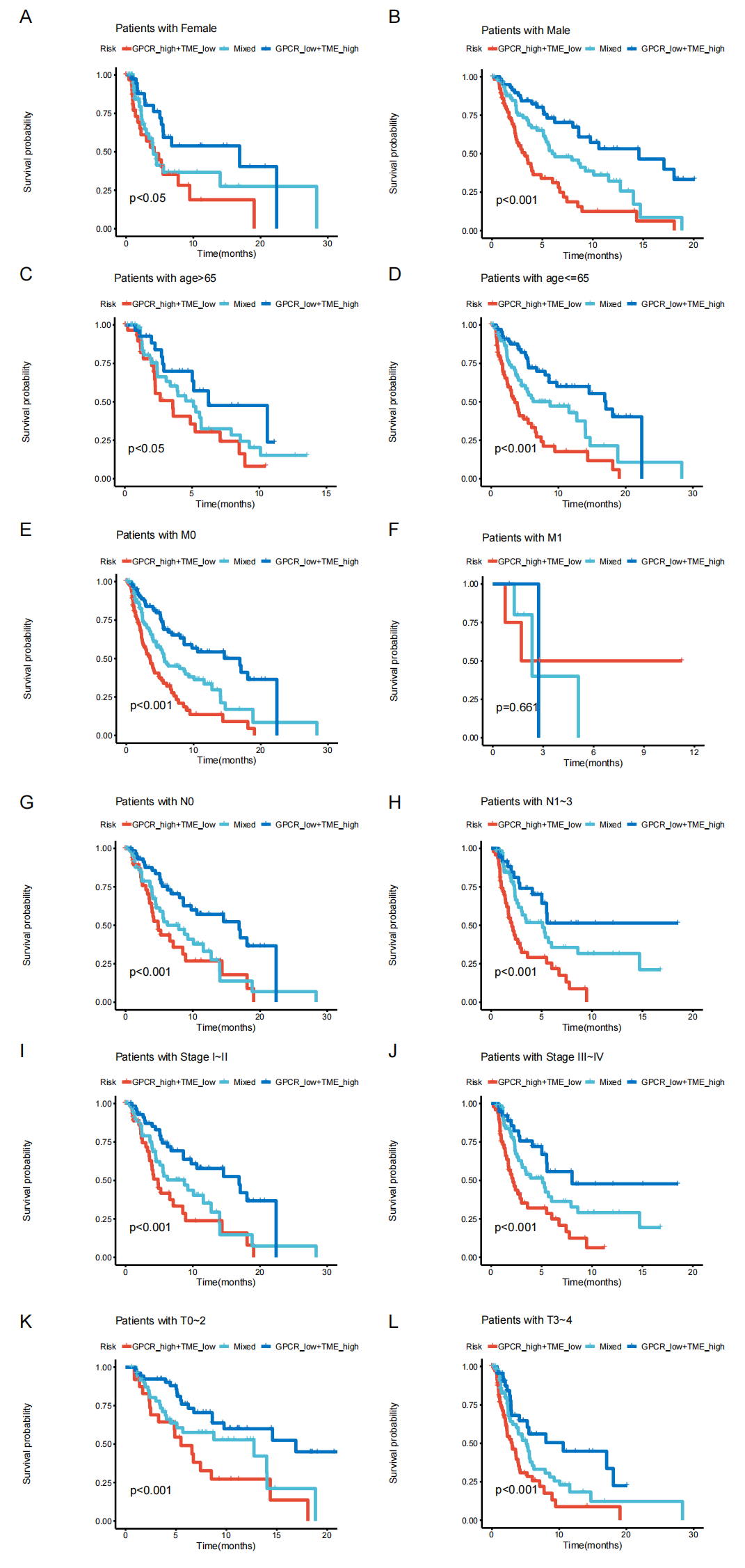

Supplement: Supplementary file 7 — Additional file 7: Figure S7. Subgroup survival analysis. A Female. B Male. C Age > 65. D Age ≤ 65. E M0. F M1. G N0. H N1–3. I Stage I−II. J Stages III−IV. K T0–2. L T3−4. [file 40001_2023_1346_MOESM7_ESM.tif]
